# Activation of mRNA translation by phage protein and low temperature: the case of *Lactococcus lactis *abortive infection system AbiD1

**DOI:** 10.1186/1471-2199-10-4

**Published:** 2009-01-27

**Authors:** Elena Bidnenko, Alain Chopin, S Dusko Ehrlich, Marie-Christine Chopin

**Affiliations:** 1 Laboratoire de Génétique Microbienne, INRA, 78352 Jouy-en-Josas, France

## Abstract

**Background:**

Abortive infection (Abi) mechanisms comprise numerous strategies developed by bacteria to avoid being killed by bacteriophage (phage). *Escherichia coli *Abis are considered as mediators of programmed cell death, which is induced by infecting phage. Abis were also proposed to be stress response elements, but no environmental activation signals have yet been identified. Abis are widespread in *Lactococcus lactis*, but regulation of their expression remains an open question. We previously showed that development of AbiD1 abortive infection against phage bIL66 depends on *orf1*, which is expressed in mid-infection. However, molecular basis for this activation remains unclear.

**Results:**

In non-infected AbiD1+ cells, specific *abiD1 *mRNA is unstable and present in low amounts. It does not increase during abortive infection of sensitive phage. Protein synthesis directed by the *abiD1 *translation initiation region is also inefficient. The presence of the phage *orf1 *gene, but not its mutant AbiD1^R ^allele, strongly increases *abiD1 *translation efficiency. Interestingly, cell growth at low temperature also activates translation of *abiD1 *mRNA and consequently the AbiD1 phenotype, and occurs independently of phage infection. There is no synergism between the two *abiD1 *inducers. Purified Orf1 protein binds mRNAs containing a secondary structure motif, identified within the translation initiation regions of *abiD1*, the mid-infection phage bIL66 M-operon, and the *L. lactis osmC *gene.

**Conclusion:**

Expression of the *abiD1 *gene and consequently AbiD1 phenotype is specifically translationally activated by the phage Orf1 protein. The loss of ability to activate translation of *abiD1 *mRNA determines the molecular basis for phage resistance to AbiD1. We show for the first time that temperature downshift also activates abortive infection by activation of *abiD1 *mRNA translation.

## Background

Bacteria have developed diverse mechanisms to avoid killing by bacteriophages (phages), which are abundant in the environment. One group of mechanisms, usually denoted as phage exclusion, or abortive infection (Abi), is characterized by a normal start of the infection process, followed by an interruption of intracellular phage development, leading to the release of few or no progeny particles and the death of the infected cell. As a consequence, further propagation of phages is prevented and the bacterial population survives.

Abi mechanisms are widespread in bacteria [1-5], but have been mainly reported in *Escherichia coli *and *Lactococcus lactis *[6-9]. The best studied mechanisms, F-factor mediated T7 exclusion, lambda Rex, Prr and Lit, all operate in *E. coli *[6,10,7]. Despite their diverse modes of action, all these systems involve a cellular protein whose function is activated or inhibited following phage infection [11-17]. Thus, Abis are considered as "altruistic death modules" that favour cell population survival following phage infection. However, recent findings suggest that Abi mechanisms might have other functions besides mediating phage resistance. The latent PrrC nuclease was shown to be induced by normal cell constituents such as pyrimidine nucleotides, which suggests that this enzyme could play roles in addition to warding off phage T4 infection [13]. PifA is suggested to be a sensor for certain environmental changes [12]. Similarly, the Rex operon could prevent programmed cell death in starved *E. coli *cells by inhibiting the ClpP family of proteases or cause a stationary phase-like response [18,19]. However, except for phage encoded proteins, no environmental signals responsible for Abi activation have been identified.

Lactococcal Abi systems have been shown to interfere with different steps of phage development, including DNA replication, maturation and packaging, transcription, capsid production and lysis of infected cells [20-22]. However, the molecular basis of these events, and the regulation of Abi systems are poorly understood. Unlike *E. coli *mechanisms, phage-dependent activation of Abis has not yet been demonstrated in lactococci. No alteration in transcriptional levels was observed for *abiA*, *abiB*, *abiD1 *and *abiG *genes examined for induction by respective phages [23-25]. A slight increase of specific transcript after phage infection was demonstrated only for *abiP *gene [26]. However, some experimental data suggests post-transcriptional regulation of expression and/or function of lactococcal *abi*s. AbiR requires an associated methylase to protect the host from its own action [21]. Cloning of intact *abiG *was shown to be lethal for heterologous *E. coli *cells [25]. Therefore, direct or indirect induction of latent Abi activity by an infecting phage or/and other factors in *L. lactis *is quite probable [20].

The AbiD1 abortive infection mechanism is encoded by a single gene, *abiD1*, of plasmid pIL105 [27,24]. Over-production of AbiD1 was shown to have bacteriostatic effect in *L. lactis *cells suggesting a tight control of *abiD1 *expression in its natural genetic background [24]. Expression of *abiD1 *was proposed to be induced following phage infection [28,20].

Among dozens of AbiD1 sensitive phages, only the small isometric-headed phage bIL66 is able to form spontaneous AbiD1-resistant mutants. These mutants have been used to study the AbiD1 mode of action [28,29]. Phage sensitivity to AbiD1 is determined by the M operon, which is expressed in mid-infection, and contains four *orfs*, denoted *orf1 *to *orf4*. The *orf3 *product is essential for phage development and its activity decreases in the presence of AbiD1 [29]. We showed that Orf3 is a structure-specific endonuclease homologous to the *E. coli *RuvC resolvase [30], which appears to be crucial for phage DNA replication and maturation prior to packaging [31,32].

All bIL66 AbiD1^R ^mutants contain a point mutation within *orf1*, the first gene of the M-operon, coding for a 42 amino acid peptide. One AbiD1^R ^mutant has an amino acid change (Orf1M1) and others have a stop codon within the first 15 residues (Orf1M2). The N-terminal region of Orf1 plays a key role in phage sensitivity to AbiD1. Phages deleted for the corresponding *orf1 *region are viable and resistant to AbiD1. However, the C-terminal region downstream of the Met15 codon is essential for phage viability and cannot be deleted from the phage genome [28]. Expression of *orf1 in trans *increases AbiD1 efficiency and prevents growth of AbiD1^R ^phage mutants. Orf1 has no known homologs in the sequence databases. We proposed that, similar to *E. coli *exclusion systems that are activated by phage-encoded proteins, Orf1 activates latent AbiD1 during phage infection, while mutant Orf1 proteins from AbiD1^R ^phages fail to do so [28]. The molecular mechanism leading to *abiD1 *activation remains to be clarified.

To gain insight into the mechanism of *abiD1 *activation and to identify potential *abiD1 *activation signals, we studied the regulation of *abiD1 *expression. Here we show that phage-encoded Orf1 protein specifically activates expression of *abiD1 *at the level of *abiD1 *mRNA translation. Moreover, expression of AbiD1 is activated at the translational level independently of phage infection during growth of cells at low temperature.

## Methods

### Bacterial strains, phages and media

*L. lactis *subsp. *lactis *IL1403 and derivatives were grown at 30°C in M17 medium supplemented with 0.5% glucose. *E. coli *TG1, BL21 (DE3) (Stratagene). *Bacillus subtilis *168 strains were grown at 37°C in LB medium. When needed, ampicillin (Ap), 100 μg ml^-1^; erythromycin (Em) (200 μg ml^-1 ^for *E. coli*, 5 μg ml^-1 ^for *L. lactis *and 0.5 μg ml^-1 ^for *B. subtilis*), and chloramphenicol (Cm) (40 μg ml^-1 ^for RNA extraction from *L. lactis *and 3 μg ml^-1 ^for *B. subtilis*) were added to the culture medium. Glucose was added to 0.5%, xylose was added to 1%. *E. coli *TG1 strain, *B. subtilis *168 strain, *L. lactis *strains IL1403, IL1403 (pIL105) [27], phage bIL66 and its AbiD1^R ^mutants bIL66M1 and bIL66M2 [29] were from our laboratory collection. Phages were enumerated as described [24].

### Molecular cloning and DNA sequence analysis

Procedures for DNA manipulation, cloning and transformation of *E. coli *were essentially as described [33]. Electrotransformation of *L. lactis *was carried out as described [34]. Polymerase chain reaction (PCR) was performed using the Gene AMP PCR System 9700 (Applied Biosystems) and ExTaq (Takara Biomedicals) essentially as described by the supplier. Nucleotide sequencing was performed on PCR products by using appropriate primers, Taq polymerase (Applied Biosystems) and fluorescent dideoxyribonucleotides on a 377A DNA sequencer (Applied Biosystems).

### mRNA extraction and analysis

Total RNA was extracted using High Pure RNA Isolation Kit (Roche) according to manufacturer's manual. Northern Blot experiments were performed using a nylon Hybond XL membrane (Amersham Pharmacia Biotech) and oligonucleotide n°1 as a probe (Additional file 1: Oligonucleotides used in this study). Oligonucleotides used for hybridization were labeled at the 5'-end with [γ-^32^P] ATP using T4 polynucleotide kinase (New England BioLabs) as described by the supplier. Northern dot-blot analysis was performed using Bio-Dot Microfiltration Apparatus (BioRad) according to instruction manual. For this, 20 μg, and successive dilutions, of RNA sample extracted from cells grown at 30°C and 18°C were treated with 50 U of RNase-free DNase I (Roche) for 20 min at 37°C and used for hybridization with *abiD1*-specific or *luxAB*-specific probe. The *abiD1 *DNA fragment was amplified using oligonucleotides n°2 and n°3. The *luxAB *DNA fragment was amplified using oligonucleotides n°4 and n°5. RNA was quantified with a PhosphorImager using ImageQuant (version 5.2; Molecular Dynamics) software. The quantitative reverse transcription-PCR was used to quantify mRNAs. Twenty μg RNA, extracted from 2-ml cultures, were treated with 50 U of RNase-free DNase I (Roche) for 20 min at 37°C. cDNAs were then synthesized using the reverse transcriptase reaction, using the CyScribe cDNA postlabelling kit (Amersham) with some modifications: RNA samples were incubated for 15 h at 42°C in a 20-μl reaction containing random nonamer primers, reverse transcriptase buffer, dithiothreitol, dNTPs, and 200 U of Superscript III reverse transcriptase (Invitrogen). Gene sequences were amplified from cDNA dilutions by PCR and quantified using an ABI Prism 7000 (Applied Biosystems). For the *abiD1 *transcript, cDNA was amplified using oligonucleotides n°6 and n°7. For the *lux *transcript, cDNA amplification was carried out using oligonucleotides n°8 and n°9. Results were normalized using the *L. lactis tuf *gene, coding for the elongation factor TU, as control. *tuf *cDNA was amplified using oligonucleotides n°10 and n°11. Changes in relative amounts of transcript mRNA normalized to *tuf *were determined using the relative C_T _method [35,36].

### Plasmid construction

Fusions between *abiD1 *translation initiation regions (TIRs) and *luxAB *genes were constructed by cloning different fragments of *abiD1 *TIR between the *Bam*HI and *Eco*RI sites of the pBluescript SKII plasmid vector (Stratagene). Then, *luxAB *genes were inserted at the ATG start position of TIRs by cloning the luciferase plasmid fusion vector pJIM1715 [37] at the *Nde*I site. Finally, pBluescript SKII DNA was deleted from the resulting plasmids by *Eco*RI digestion and self-ligation. Different *abiD1 *TIR DNA fragments were amplified using pIL105 plasmid DNA, oligonucleotide n°13 carrying *Bam*HI and *Nde*I sites, and one of the following oligonucleotides: n°14, carrying an *Eco*RI site (fragment I), n°15 carrying an *Eco*RI site (fragment II), n°16 carrying an *Eco*RI site (fragment III). The *abiD1 *TIR IV fragment was amplified using fragment II cloned in pBluescript SKII plasmid vector as a template, oligonucleotides n°17, carrying an *Eco*RI site and n°18, carrying a *Cla*I site. Resulting plasmids were designated pIL5014 (*abiD1 *TIR I), pIL5015 (*abiD1 *TIR II), pIL5016 (*abiD1 *TIR III) and pIL5026 (*abiD1 *TIR IV). The *orf1 *gene from phage bIL66 and bIL66M1 or bIL66M2 mutants was inserted upstream of the different *abiD1 *TIR:*luxAB *fusions. For this, PCR fragments corresponding to the different *orf1 *genes were first cloned in the pBluescript SKII plasmid vector. Fragments were recovered by digestion with *Eco*RI, and then cloned at the *Eco*RI site of plasmids pIL5014 and pIL5015. Oligonucleotides n°19 and n°20 were used for the PCR amplification. *aldB:luxAB *fusion plasmid pIL5032 was constructed in the same manner using oligonucleotides n°21 and n°22, carrying *Eco*RI and *Bam*HI (n°21), and *Nde*I sites (n°22). To construct plasmid pIL5033, the *orf1 *gene from phage bIL66 was inserted at the *Eco*RI site of pIL5032. To integrate the *orf1 *gene from phage bIL66 or bIL66M2 into the *B. subtilis *chromosome, both genes were initially cloned into *Pac*I, *Bam*HI sites of plasmid pSWEET [38]. To do this, *orf1 *or *orf1M1 *PCR fragments were amplified with oligonucleotides n°23 carrying a *Pac*I site and n°24 carrying a *Bam*HI site. Cloning was performed in *E. coli *strain TG1. The resulting plasmids were targeted to *amyE *of *B. subtilis via *double recombination as linear DNA, using *Pst*I-digested plasmid. To construct plasmid pIL5033, the *rbfA *gene from *L. lactis *IL1403 was amplified with oligonucleotide n°25, carrying a *Sal*I site, and n°26, carrying a *Pst*I site, and ligated to *Sal*I, *Pst*I-digested plasmid pGKV259 [39].

### Luciferase assay

*L. lactis *IL1403 cells transformed with various *luxAB *constructs were grown to an optical density at 600 nm (OD_600_) of 0.4 at either 30°C or 18°C in M17 medium. Experimental conditions for oxidative stress and acid pH used for the luciferase assay were mainly as described [40,41]. Exponentially growing *L. lactis *IL 1403 cells were incubated with 1 mM H_2_O_2 _to generate oxidative stress conditions. The range of acid pH tested was 4.5, 4.2 and 4.0. *B. subtilis *168 cells were grown to an OD_600 _of 0.1 at 30°C in LB medium in the presence of glucose, washed in LB and grown to an OD_600 _of 0.4 at 30°C in LB medium in the presence of xylose. To measure luciferase activity, 1 ml of culture was mixed with 5 μl of nonylaldehyde (Acros Organics), and the light emission was measured immediately in a Lumat LB9501 luminometer (Berthold).

### Western blotting

The 3 × FLAG peptide sequence was cloned in the *Nde*I site of *abiD1 *TIR I:*luxAB *fusion plasmid pIL5014. The DNA fragment carrying the 3 × FLAG peptide sequence was obtained by PCR amplification using p3 × FLAG-CMV-7 expression vector (Sigma-Aldrich) as a template and oligonucleotides n°29 and n°30 each carrying an *Nde*I site. The final construct was verified by sequencing. *L. lactis *cells carrying FLAG tagged *abiD1 *TIR I:*luxAB *fusion plasmid were grown to an OD_600 _of 0.4 at either 30°C or 18°C in M17 medium medium supplemented with 0.5% glucose. Cells were harvested, resuspended in 20 mM Tris-HCl, 10 mM EDTA buffer, and lysed with 4 mg/ml lysozyme (Sigma-Aldrich) at 37°C 20 min. Equal amounts (20 mg) of proteins were separated on 8% SDS-PAGE gel electrophoresis and transferred to nylon Hybond-P membrane (Amersham Pharmacia Biotech). The membrane was blocked in Tris-buffered saline-0.1% Tween 20 with 5% nonfat milk overnight and afterwards incubated sequentially with anti-FLAG tag mouse monoclonal antibody M2 (dilution 1:5000, Sigma -Aldrich) for 3 h and then alkaline phosphatase-conjugated goat anti-mouse IgG (dilution 1: 20000, Sigma-Aldrich) for 1 h. Immunolabeled proteins were revealed using ECL Plus Western Blotting Detection System (Amersham Pharmacia Biotech) according to manufacturer's manual. Proteins were quantified with a PhosphorImager using the ImageQuant software.

### Production and purification of Orf1 protein

Protein expression and purification were performed using the IMPACT-SN system (New England Biolabs). The *orf1 *gene from phage bIL66 and bIL66M1 was cloned in the pTYB11 expression vector. The DNA fragment carrying *orf1 *was obtained by PCR amplification using phage DNA as a template and oligonucleotides n°31 (for Orf1 and Orf1M1 proteins) or n°32 (for shortened Orf1M2 protein), both carrying a *Sap*I site and n°33, carrying an *Eco*RI site. Final constructs with 5' intein-tagged *orf1 *genes were controlled by sequencing. Expression was performed in BL21 (DE3) *E. coli *cells at 17°C upon 15 h of induction with 0.6 mM IPTG. Proteins extracted from the soluble fraction were further cleaved from the intein part and purified as recommended by the supplier. Purity of the final Orf1 protein preparation, evaluated using the Novex NuPage Pre-cast Gel System (Invitrogen), was > 90%. The first eight N-terminal amino acids (MTEEQLLF) were confirmed by MALDI-TOF (Applied Biosystems, Voyager DE super STR) to be identical to those deduced from the nucleotide sequence: The molecular mass of purified protein was 4850.43 Da and corresponded to the theoretical molecular mass (4808.5 Da) of Orf1. Orf1M1 and Orf1M2 proteins were purified in the same manner.

### RNA binding experiments

*abiD1*, *aldB, trpA, osmC *and M-operon RNA transcripts were prepared by run-off transcription of DNA templates with T7 RNA Polymerase transcription kit (Stratagene) in the presence of [α^32^P] rUTP (800 Ci/mmol; MP Biomedicals) according to manufacturer's manual. The different *abiD1 *DNA templates were obtained by PCR with the following oligonucleotides carrying the T7 promoter sequence: n°34 (*abiD1 *transcript 1 and *abiD1 *transcript 5), n°35 (*abiD1 *transcript 2), n°36 (*abiD*1 transcript 3), n°37 (*abiD1 *transcript 4), n°38 (*abiD1 *transcript 6), n°39 (*abiD1 *transcript 7), and used with oligonucleotides n°40 (*abiD1 *transcripts 1 to 4), n°41 (*abiD1 *transcript 5), n°42 (*abiD1 *transcript 6) and n°43 (*abiD1 *transcript 7). The DNA template for *L. lactis *IL1403 *osmC *RNA transcript was obtained by PCR with oligonucleotides: n°44 and n°45. The DNA template for *L. lactis *IL1403 *aldB *RNA transcript was obtained by PCR with oligonucleotides n°46 and n°47. The DNA template for *L. lactis *IL1403 *trpA *RNA transcript was obtained by PCR with oligonucleotides n°48 and n°49. The DNA template for bIL66 M-operon RNA transcript was obtained by PCR with oligonucleotides n°50 and n°51. The labelled RNA transcripts were purified by elution after separation on a non-denaturing 6% polyacrylamide gel at room temperature. Purified RNAs were resuspended in binding buffer (10 mM Tris pH 7.5, 21 mM KCl, 1 mM EDTA) and renatured by heating to 90°C for 1 min and chilling on ice for 2 min. Binding reaction mixture, containing 10 ng of the labeled transcript and various concentrations of purified Orf1 protein in 10 mM Tris pH 7.5, 21 mM KCl, 1 mM EDTA, 1 mM DTT, 5% glycerol, was incubated at 20°C for 30 min. Reactions were analyzed by electrophoresis in an 8% polyacrylamide gel at room temperature.

## Results

### Transcriptional analysis of *abiD1*

**Figure 1:**
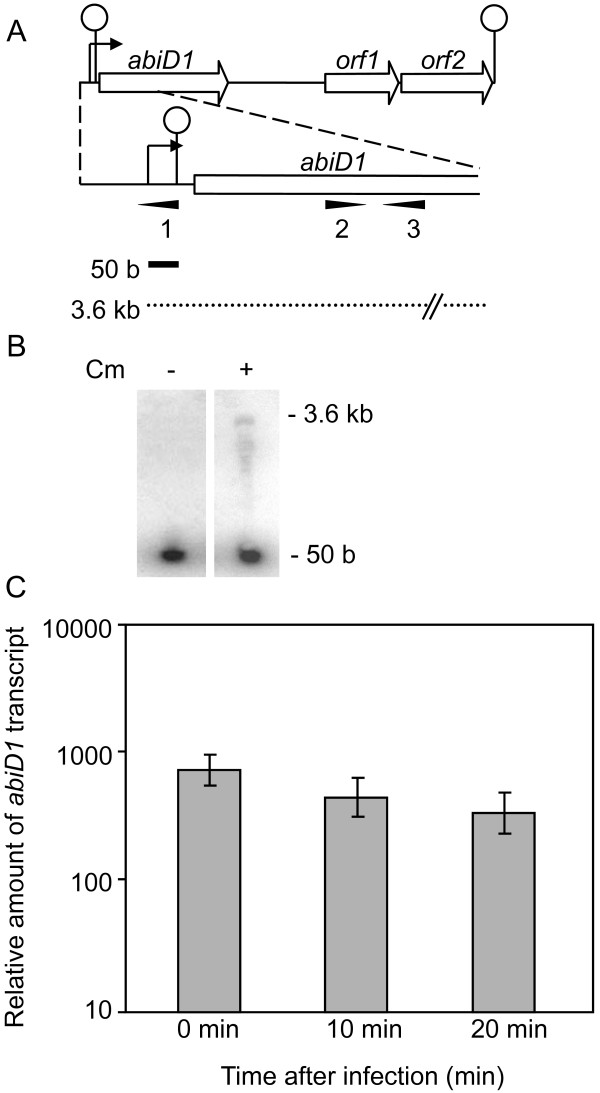
**Transcriptional analysis of the *abiD1 *gene**. (A) Schematic organization of the *abiD1 *gene region. Bent arrows and circles denote promoter and terminator sequences, respectively. Position of oligonucleotides used for Northern hybridization (1) and quantitative RT-PCRs are indicated by tail-less arrows. Transcripts initiated at the *abiD1 *promoter are shown by solid (50 b) and broken (3.6 kb) lines. (B) Northern hybridization results. RNA was extracted from IL1403 AbiD1+ cells grown with or without Cm. Hybridization was performed with oligonucleotide n°1. (C) Quantitative RT-PCRs. *abiD1 *transcript was followed in IL1403 AbiD1+ cells. Samples were taken before (time 0), 10 and 20 min after infection with bIL66 phage. Amount of *abiD1 *transcript was normalized to *L. lactis tuf *transcript level. Values shown are means of 9–15 measurements, expressed in arbitrary units.

To investigate the regulation of *abiD1 *gene expression, we first examined the *abiD1 *transcriptional unit. Sequence analysis of plasmid pIL105 (8506 bp, GenBank accession number AF116286) suggested that *abiD1 *might be transcribed in an operon with two downstream genes, coding for putative proteins of unknown function (Fig. 1A). A putative terminator sequence was identified 3.6 kb downstream of the determined transcription start [24]. However, previous attempts to visualize a full-length *abiD1 *transcript in Northern experiments were unsuccessful. The only visible transcript initiated at *abiD1 *promoter was 50 b long and was shown to stop at a terminator structure localized just upstream of the *abiD1 *gene [24] (Fig. 1B). Full-length *abiD1 *transcript was not revealed by Northern blot during abortive infection of IL1403 AbiD1+ cells with sensitive phage bIL66 [24]. Taking into account the pronounced abortive infection phenotype of IL1403 AbiD1+ cells, we hypothesized that during phage infection, full-length *abiD1 *transcript is present in functional amounts, but is probably highly unstable. To overcome this putative instability, we tried to visualize the full-length *abiD1 *transcript by extracting RNA in the presence of chloramphenicol (Cm), which is known to stabilize bacterial mRNAs [42]. Two transcripts of 50 b and approximately 3.6 kb were revealed upon hybridization with primer 1, the larger being much less abundant that the smaller (Fig. 1B). This 3.6-kb *abiD1 *transcript was not observed when RNA was extracted in the absence of Cm, thus confirming low stability of *abiD1 *mRNA.

These results, together with those published earlier [24], indicate that the low amounts of the 3.6 kb mRNA transcript is most likely due to activity of the transcriptional terminator located upstream of the *abiD1 *gene. Read through across this terminator would be responsible for expression of *abiD1*. To investigate the possibility that phage-encoded protein(s) might increase synthesis of the full-length 3.6 kb *abiD1 *transcript either by an anti-termination mechanism or by stabilization of specific mRNA, we studied the synthesis of *abiD1 *mRNA after phage infection using quantitative reverse transcription PCR (QRT-PCR) technique. The amount of the full-length *abiD1 *transcript was determined during one cycle of phage bIL66 multiplication, which takes approximately 40 min. RNA samples were taken immediately before (time 0), in the middle (10 min) and at the beginning of the late (20 min) steps of phage infection [43]. Our results show that the amount of full-length *abiD1 *transcript varied little during phage infection, and decreased slightly with time (Fig. 1C). Taken together, these results indicate that *abiD1 *is co-transcribed with two other genes of unknown function. The unstable full-length 3.6 kb *abiD1 *transcript results from read through across the transcriptional terminator and is present in low amounts, which do not increase during abortive infection with sensitive phage. This suggests that activation of the AbiD1 mechanism is not exerted at the level of synthesis or stabilization of *abiD1 *full-length mRNA.

### Analysis of *abiD1 *expression at the translational level

**Figure 2:**
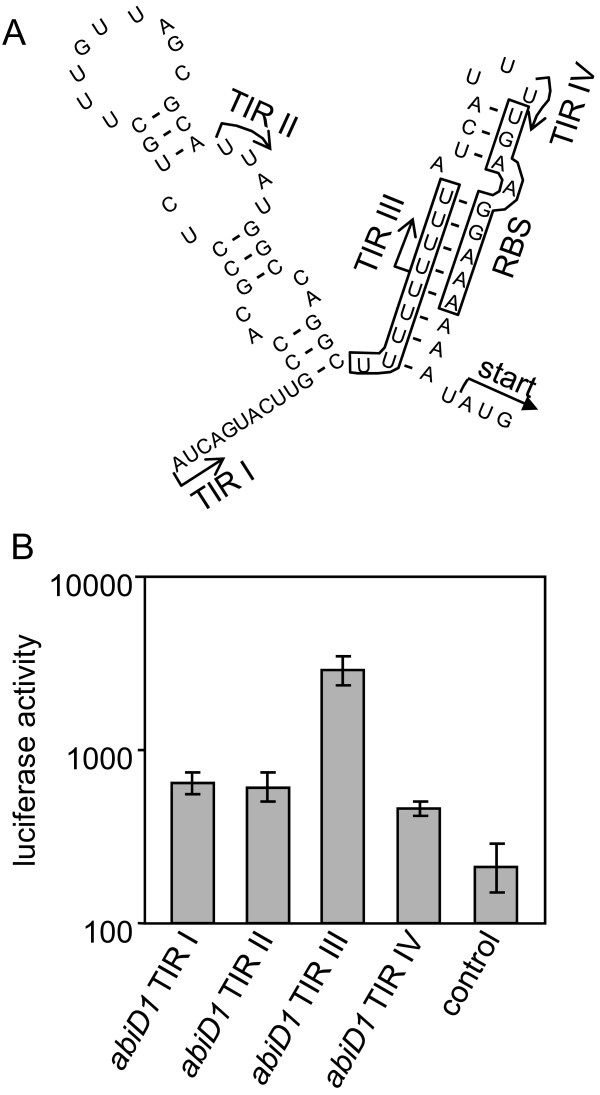
**Translation of the *abiD1 *mRNA**. (A) MFOLD [44] predicted secondary structure of the *abiD1 *translation initiation region (TIR). The sequence is presented from the transcriptional start (+ 1) to the *abiD1 *AUG start codon. Ribosome binding site (RBS) and the poly-U stretch are boxed. Arrows show the 5' ends of different TIRs used. The start of *luxAB *gene in fusion constructs is indicated. (B) Translation of *abiD1 *TIR:*luxAB *fusions. The background level of luciferase activity was measured in the presence of plasmid pJIM1715 [50]. Experiments were performed at 30°C. Results are means of 6–10 independent experiments. Luciferase activity is shown in arbitrary light units (lux/OD unit at an OD_600 _of 0.4).

Expression of *abiD1 *was examined at the translational level. Translation initiation region (TIR) of *abiD1 *mRNA is 76 nucleotides long, and contains a transcriptional terminator, and a ribosome binding site (RBS) UUUGAAGG complementary to the *L. lactis *16S rRNA sequence with one mismatch (underlined) [44]. The RBS is preceded by a poly-U sequence, which is a part of the transcription terminator (Fig. 2A). Analysis of the *abiD1 *TIR sequence using MFOLD version 3.1 suggests the existence of a stem-loop structure (ΔG = -9.1 Kcal/mole) immediately downstream of the transcriptional terminator [45]. This structure could potentially sequester two elements known to positively control translation initiation efficiency, namely the RBS sequence and the poly-U sequence, which provides a binding site for ribosomal S1 protein [46]. Predicted by MFOLD mRNA secondary structure was cross-validated by the programs RNAfold and Kinefold [47-49].

To test *abiD1 *translation efficiency we used the *luxAB *reporter gene from *Vibrio fischeri *[50]. Four DNA fragments corresponding to different parts of *abiD1 *TIR but missing the *abiD1 *promoter sequence were fused to the ATG start codon of the *luxAB *reporter gene that lacked its translational signals [[37], Methods]. Translation of *luxAB *is thus dependent on initiation signals carried on the cloned *abiD1 *TIR fragment. The constructs were transcribed from a constitutive plasmid promoter [37]. The four cloned fragments ended at the *abiD1 *ATG site and differed at their 5' ends (Fig. 2A). TIR I starts at the + 1 transcription initiation point and would be able to form two stem-loop mRNA structures. TIR II contains a truncated transcriptional terminator stem-loop and an RBS-sequestering stem-loop mRNA. TIR III contains a shortened poly-U sequence and would most probably not sequester the RBS. TIR IV contains only the *abiD1 *RBS sequence. Resulting plasmids were tested for their capacity to direct luciferase synthesis in *L. lactis *IL1403 cells. Luciferase activities detected with TIR I, II or IV were weak (Fig. 2B). Removal of the transcriptional terminator sequence (TIR II) has no positive effect on luciferase activity, confirming that it does not play a major role in regulating *abiD1 *expression. Luciferase activity detected with TIR III was 4.5-fold higher, suggesting that sequestering of *abiD1 *RBS by secondary structure has a negative effect on translation efficiency. Nevertheless, the level of luciferase activity directed by *abiD1 *TIR III was relatively low: A control, the lactococcal *aldB *RBS cloned on plasmid vector pJIM1715, which reached 180 ± 30 × 10^3 ^arbitrary light units (lux/OD unit at an OD of 0.4) [37], while *abiD1 *expression in the same context was ~100-fold lower. The difference observed between TIR III and TIR IV suggests a possible role of the poly-U sequence in increasing translation initiation, similar to what was described in *E. coli *[51]. Taken together, these results indicate that translation of *abiD1 *mRNA is inefficient and suggest that some *trans*-acting factor(s) might be required for its activation.

### Phage Orf1 activates translation of *abiD1 *mRNA

**Figure 3:**
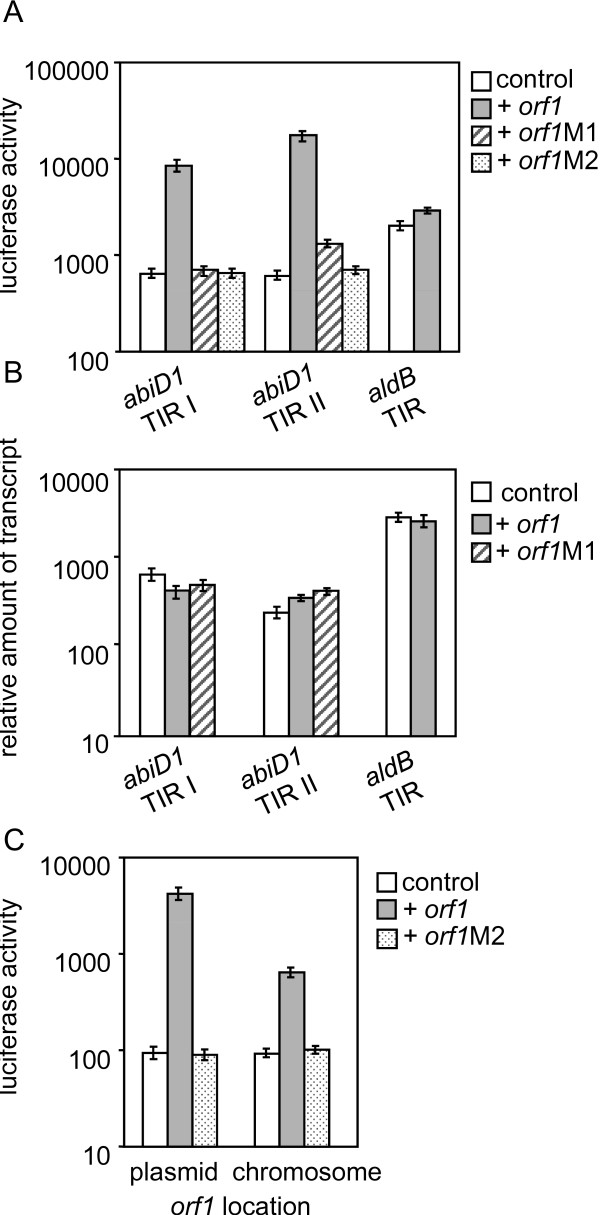
**Orf1 activates translation of *abiD1 *TIR: *luxAB *fusions**. (A) Translation of *abiD1 *TIR I:*luxAB*, *abiD1 *TIR II:*luxAB *and *aldB *TIR:*luxAB *fusions in the absence (control) or in the presence of *orf1*, *orf1*M1 and *orf1*M2 genes in *L*. *lactis *IL1403 cells. Experiments were performed at 30°C. Results are means of 6 independent experiments. (B) Transcription of *abiD1 *TIR:*luxAB *fusions. The amount of *abiD1 *TIR I: *luxAB *and *abiD1 *TIR II: *luxAB *transcripts was measured by quantitative RT-PCR in the absence (control) and in the presence of *orf1 *or *orf1M1 *genes in *L. lactis *cells grown at 30°C. The *aldB *TIR: *luxAB *fusion was used as a control. Amount of transcript was normalized to the *L. lactis tuf *transcript level. Values are means of 7 – 15 measurements, expressed in arbitrary units. (C) Translation of *abiD1 *TIR II:*luxAB *fusion in the presence of plasmid- or chromosome- integrated *orf1 *and *orf1*M2 in *B. subtilis *168 cells. Experiments were performed at 30°C. Results are means of 4–5 independent experiments. Luciferase activity is shown in arbitrary light units (lux/OD unit at an OD_600 _of 0.4).

In the search for putative factors activating translation of *abiD1 *mRNA we gave special attention to the phage-encoded Orf1 protein, whose expression in mid-infection is essential for bIL66 abortive infection [28,29]. To explore the possibility that Orf1 protein acts as an activator of *abiD1 *expression, we studied the capacity of *abiD1 *TIR to direct *luxAB *translation in the presence of Orf1. To keep a constant ratio between *orf1 *and *abiD1 *TIR in the different constructs, we cloned *orf1 *on plasmids carrying *abiD1 *TIR:*luxAB *translational fusions, so that *orf1 *and *abiD1 *TIR:*luxAB *were both transcribed from the same constitutive plasmid promoter. The fusion of *luxAB *reporter gene with TIR of *L. lactis aldB *gene, preceded by a stable secondary structure that trapped the *aldB *RBS [52] was used as control. All plasmids were tested for luciferase activity in IL1403 cells. The presence of *orf1 *caused a 13- to 27-fold increase in luciferase production, depending on the *abiD1 *TIRs (Fig. 3A). We replaced phage *orf1 *gene by mutated *orf1 *alleles M1 (A35D) or M2 (Q3*), coding for Orf1 protein with an amino acid substitution or deletion of the N-terminal part, respectively. Both mutations render bIL66 phage resistant to AbiD1 and differ from *orf1 *by one nucleotide [28]. Presence of mutated *orf1 *genes abolished the increase of luciferase activity (Fig. 3A, TIRI and TIR II).

The level of luciferase activity directed by the control *aldB *TIR:*luxAB *fusion was not increased in the presence of the *orf1 *gene (Fig. 3A). These results indicate that *orf1 *specifically increases expression of *abiD1*. To measure the amount of the specific transcript, we analyzed *luxAB *mRNA using QRT-PCR. Similar amounts of *luxAB *RNA were detected in the presence and in the absence of either *orf1 *or *orf1M1 *gene for all tested constructs (Fig. 3B). These results indicate that the effect of *orf1 *on *abiD1 *TIR:*luxAB *fusions is not exerted on the transcriptional level.

We also performed Northern analysis of Cm-stabilized *abiD1 *specific mRNA extracted from IL1403 AbiD1^+ ^cells in the presence or absence of *orf1*-expressing plasmid pIL2002, which is known to increase AbiD1 activity [28]. As shown in Additional file 2 (Transcriptional analysis of the *abiD1 *in the presence of phage *orf1 *gene), the 3.6 kb transcript was detected in equal amounts in the presence or in the absence of Orf1w^+^.

As transcription-translation reactions are known to be highly conserved among bacterial species [53], we used *B. subtilis *168 cells to test *orf1 *activity in *trans*. First we measured luciferase activity directed by plasmid constructs in the presence or absence of *orf1 *in *cis*. Luciferase activity directed by *abiD1 *TIRII:*luxAB *in *B. subtilis *cells was 45-fold higher in the presence of *orf1 *compared to a control plasmid carrying *abiD1 *TIRII:*luxAB *without *orf1*. The *orf1*M2 did not increase translation of *abiD1 *TIR II:*luxAB *fusion (Fig. 3C). To test *orf1 *activity in *trans*, *orf1 *or its mutant *orf1*M2 allele were placed under the control of inducible *B. subtilis *P*xyl *promoter and integrated at the *amyE *locus of *B. subtilis *chromosome. Luciferase activity directed by *abiD1 *TIR II:*luxAB *fusion was measured at 30°C in the presence of 1% xylose. In these conditions, translation of *abiD1 *TIR II:*luxAB *fusion was activated approximately 7-fold in the presence of *orf1 *compared to the control. Activation was not observed with *orf1*M2 (Fig. 3C). Therefore, *orf1 *activates expression of *abiD1 *TIR:*luxAB *fusion in *trans *and *in cis *in the heterologous *B. subtilis *host. Taken together, our results indicate that *orf1 *positively regulates translation of the *abiD1 *mRNA. The activation function of *orf1 *is most probably specific, as it was abolished by mutations rendering phage resistant to AbiD1, and it was not observed with control *aldB *TIR:*luxAB *fusion construct.

### Low temperature activates translation of *abiD1 *mRNA and abortive infection phenotype

**Figure 4:**
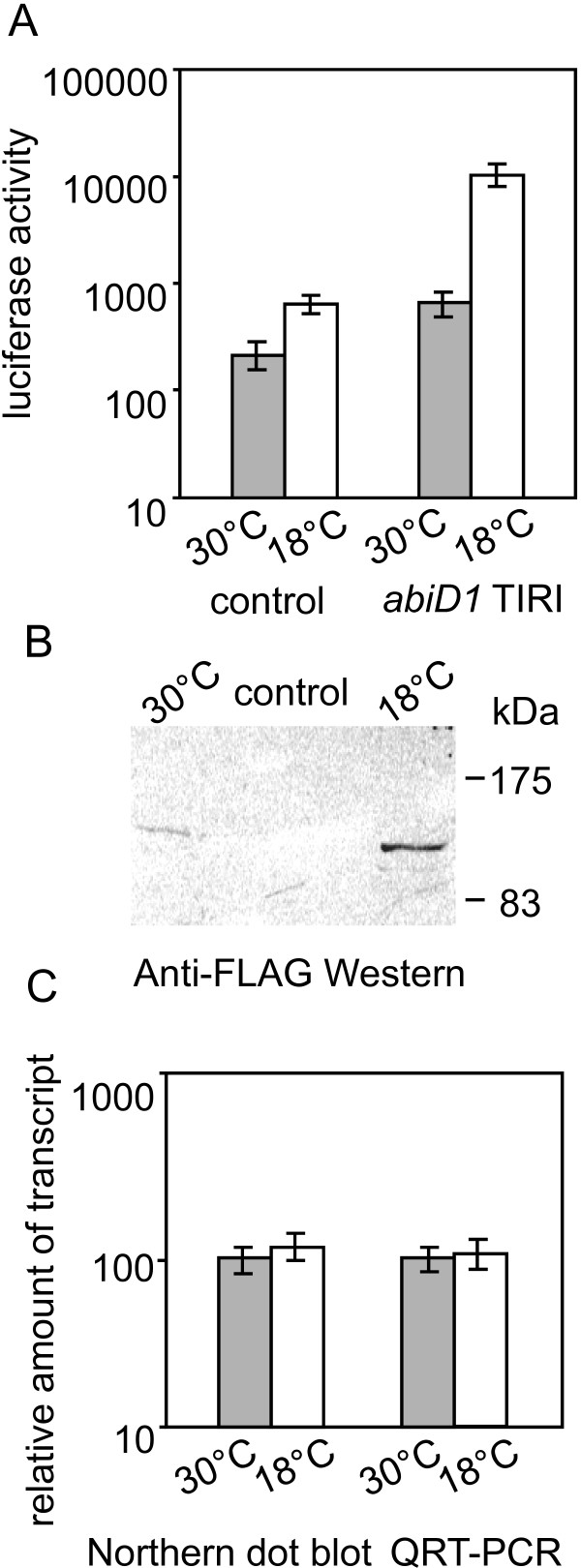
**Effect of temperature on expression of the *abiD1 *TIRI : *luxAB *fusion**. (A) Translation of *abiD1 *TIR I:*luxAB *fusion in *L*. *lactis *IL1403 cells at 30°C and 18°C. The background level of luciferase activity at 30°C and 18°C was measured in the presence of the control plasmid pJIM1715 [50]. Results are means of 5 independent experiments. Luciferase activity is shown in arbitrary light units (lux/OD unit at an OD_600 _of 0.4). (B) Expression of FLAG-tagged LuxAB at 30°C and 18°C. *L. lactis *cells carrying FLAG tagged *abiD1 *TIR I:*luxAB *fusion plasmid were grown to an OD_600 _of 0.4 at either 30°C or 18°C in M17 medium. Twenty mg of total cell protein were separated on an 8% SDS-PAGE gel. Western blot was developed with anti-FLAG tag M2 monoclonal antibody. The control panel shows cell lysates without specific FLAG-tagged protein. Molecular masses of standard proteins (Prestained Protein Marker, Broad Range, New England BioLabs) are indicated on the right. (C) Transcription of *abiD1 *TIR:*luxAB *fusion. The amount of *abiD1 *TIR I: *luxAB *transcript in *L. lactis *cells grown at 30°C and 18°C was quantified by ImageQuant software after dot-blot Northern hybridization and by quantitative RT-PCR. Values are means of 3–7 measurements, expressed in arbitrary units.

The above results indicated that expression of *abiD1 *is activated by the infecting bIL66 phage. In a search for other potential extracellular induction signals, we measured the activity of *abiD1 *TIR I:*luxAB *fusion in different cell growth conditions. Various types of stress to which *L. lactis *is exposed during normal growth (acid pH, oxidative stress, and low temperature) were tested. Among these, growth of IL1403 *abiD1 *TIR I:*luxAB *cells at the temperature range of 18°C to 20°C was found to increase luciferase activity about 15-fold as compared to 30°C (Fig. 4A). This level of luciferase activity was reached after 2.5 hours incubation of IL1403 *abiD1 *TIR I:*luxAB *cells at 18°C. However, we also detected a 3-fold increase of the background luciferase level in cells carrying the control *luxAB *plasmid pJIM1715 at 18°C, a phenomenon observed previously and attributed to more efficient folding of the LuxAB protein at low temperature [54-56]. Correcting for this increase, growth at 18°C conferred 5-fold higher expression of *luxAB *when fused to the *abiD1 *TIR I region (Fig. 4A).

To confirm cold-induced activation of expression initiated by *abiD1 *TIR at the protein level we constructed in-frame fusions of *luxAB *with FLAG tag sequence on *abiD1 *TIR I:*luxAB *plasmid pIL5014. *abiD1 *TIR I directed synthesis of tagged luciferase at 30°C and 18°C was analyzed by Western blotting with anti-FLAG antibodies. The amount of LuxAB-FLAG protein produced at 18°C was approximately 6-fold higher than at 30°C (Fig. 4B). Thus, the corrected level of specific luciferase activity detected at 18°C corresponds well to the increased level of protein synthesis at this temperature, and not to more efficient LuxAB folding. The amount of *luxAB *mRNA was measured by QRT-PCR and dot-blot Northern hybridization. Both methods revealed similar amounts of *luxAB *RNA in cells grown at 30°C and 18°C (Fig. 4C). These results indicate that increase of luciferase activity of *abiD1 *TIR I:*luxAB *fusion at 18°C is not due to activation of *luxAB *transcription. No additional increase of luciferase activity was observed in the presence of Orf1 at 18°C (data not shown). These results suggest that *abiD1 *expression is increased in *L. lactis *cells grown at 18°C, and that induction occurs at the translational level.

These results led us to measure efficiency of phage abortive infection at 18°C. Phage bIL66 and two AbiD1^R ^bIL66 mutants (bIL66 M1 and bIL66 M2) were plated on IL1403 and IL1403 AbiD1^+ ^cells grown at 30°C and 18°C (Table 1). At 30°C phage bIL66 formed spontaneous AbiD1^R ^mutants at a frequency of 10^-5 ^and the two bIL66 mutants grew normally. At 18°C, in the presence of AbiD1, spontaneous AbiD1^R ^bIL66 mutants were not observed and growth of the two resistant mutants was strongly inhibited. In the absence of AbiD1, plaque-forming efficiency and plaque morphology of the wild type and mutant phages were similar at 18°C and at 30°C. These results confirm that the AbiD1 phenotype is strongly activated during cell growth at low temperature independently of phage infection. Therefore, AbiD1 fits into the group of *L. lactis *cold shock-inducible proteins.

**Table 1:** AbiD1 abortive infection is activated at 18°C

		Phage titre^*a *^(PFU/ml)^*b*^		
Temperature:	30°C		18°C	

Strain :	IL1403	IL1403 AbiD1^+^	IL1403	IL1403 AbiD1^+^

Phage :				
				
bIL66	3 × 10^10^	2 × 10^5*c*^	2 × 10^10^	< 10^2^
		3 × 10^3 ^t		
bIL66.M1	1 × 10^9^	8 × 10^8^	1 × 10^9^	10^2 ^t
bIL66.M2	7 × 10^9^	6 × 10^9^	7 × 10^9^	10^2 ^t

^*a *^Average of three independent experiments.

^*b *^PFU: plaque forming units.

^*c*^clear plaques formed by spontaneous AbiD1 resistant mutants.

t, very small, turbid plaques.

### *L. lactis *RbfA protein inhibits activation of AbiD1 phenotype by low temperature, not by Orf1 protein

Overproduction of cold shock protein RbfA was shown to accelerate growth adaptation of *E. coli *at low temperature and greatly decreases the adaptation period following cold shock [57-60]. The RbfA protein is conserved in most prokaryotic organisms [61].

To test whether high levels of RbfA protein weaken the AbiD1 phenotype activated either by phage encoded protein Orf1 or by low temperature we cloned the *L. lactis rbfA *gene in high copy plasmid pGKV259 under the control of a strong constitutive promoter. The construct was introduced in the IL1403 AbiD1+ cells and abortive infection efficiency was determined at 30°C and 18°C (Table 2). RbfA had no effect on phage development at 30°C. Interestingly, growth of the bIL66M1 AbiD1^R ^phage on IL1403 AbiD1+ cells carrying the RbfA plasmid was as efficient at 18°C as at 30°C. In the absence of RbfA, bIL66 M1 grew poorly at 18°C due to activation of abortive infection by low temperature (Table 2). This indicates that in the absence of active Orf1 protein synthesis of the AbiD1 protein was not induced by low temperature because of the presence of RbfA. In contrast, growth of wild type bIL66 phage was not detected at 18°C in the absence of *rbfA *and was inefficient in the presence of *rbfA*, indicating that synthesis of AbiD1 is still activated in both strains by wild type Orf1 protein (Table 2). These results show that overproduction of RbfA (i) does not affect induction of AbiD1 abortive infection by the phage Orf1, intact in the wild-type phage, (ii) abolishes induction of AbiD1 abortive infection by low temperature, favouring the notion that *abiD1 *is a cold shock- regulated gene.

**Table 2:** *L. lactis *RbfA protein inhibits activation of AbiD1 phenotype by low temperature

		Phage titre^*a *^(PFU/ml)^*b*^		
Temperature:	30°C		18°C	

		IL1403 AbiD1^+^		
Strain^*a *^:				
	-	+ *rbfA*	-	+ *rbfA*

Phage :				
				
bIL66	8 × 10^4*c*^	2 × 10^5*c*^	<10^2^	3 ×10^3^t
	2 × 10^3 ^t	3 × 10^3 ^t		
bIL66.M1	4 × 10^8^	3 × 10^8^	2 × 10^2 ^t	3 ×10^8^

^*a *^Average of three independent experiments.

^*b *^IL1403 AbiD1^+ ^strain carrying either pGKV259 vector (-) or pGKV259:*rbfA *(+ *rbfA*)

^*c*^clear plaques formed by spontaneous AbiD1 resistant mutants.

t, very small, turbid plaques.

### *Orf1 *binds *abiD1 *mRNA

Our study identified two AbiD1 activating signals, the phage-encoded protein Orf1 and low temperature, both acting at the level of mRNA translation. The Orf1 activation pathway differs from the cold shock pathway as it was shown not to be susceptible to RbfA-mediated cell adaptation to growth at low temperature. We supposed that Orf1 might activate translation of *abiD1via *binding to *abiD1 *mRNA.

**Figure 5:**
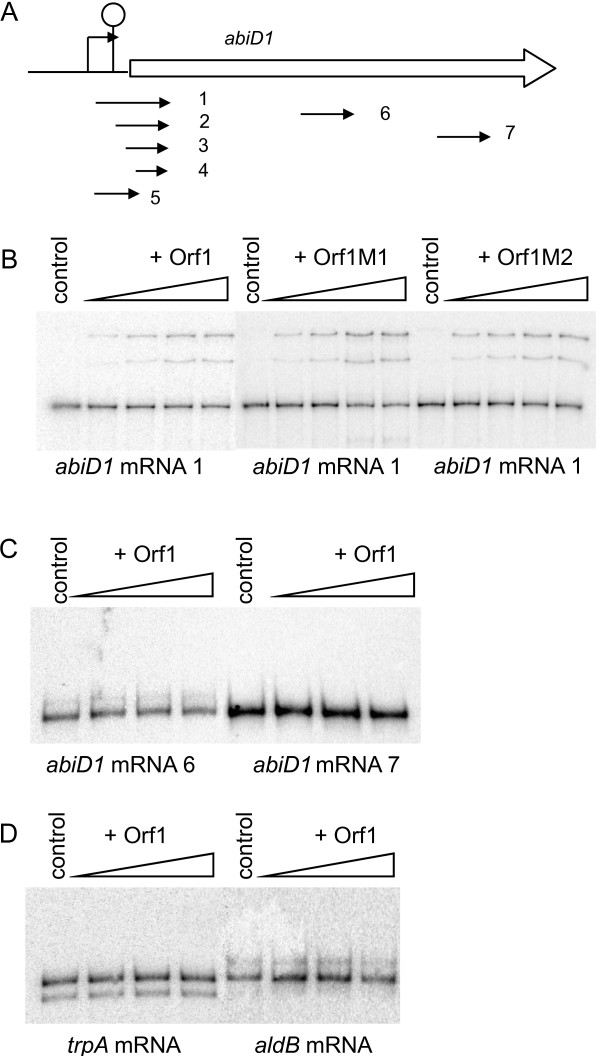
**Orf1-RNA binding activity**. (A) Schematic organization of the *abiD1 *gene. Arrows indicate position of *abiD1 *transcripts (1 to 7) used for binding experiments. (B) Binding of purified Orf1, OrfM1 and Orf1M2 to the *abiD1 *mRNA 1. Ten ng of radiolabelled *abiD1 *mRNA (transcript 1) were incubated with increasing amounts of Orf1, OrfM1 or OrfM2 protein (0; 0.03 μM; 0.05 μM; 0.11 μM; 0.22 μM), followed by separation of the nucleoprotein complex in an 8% non-denaturing polyacrylamide gel at room temperature. (C) Binding of Orf1 to the *abiD1 *mRNA 6 and *abiD1 *mRNA 7. (D) Binding of Orf1 to *trpA *mRNA and *aldB *mRNA. Experiments (C and D) were performed with increasing amounts of Orf1 protein (0; 0.11 μM; 0.22 μM; 0.33 μM) in the same conditions as above.

To examine this hypothesis we tested whether Orf1 can specifically bind TIR of *abiD1 *mRNA. For this purpose, we performed a gel mobility shift assay using purified Orf1 protein and an *abiD1 *transcript that was synthesized in *vitro *with phage T7 RNA Polymerase. The transcript, designated mRNA 1, starts at the + 1 transcription initiation point and is 297 nucleotides long (Fig. 5A). Incubation of mRNA 1 with increasing amounts of Orf1 protein followed by electrophoresis in non-denaturing polyacrylamide gel led to the appearance of bands with reduced mobility in the gel indicating the formation of RNA-protein complexes (Fig. 5B). The presence of several retarded bands reflects either formation of ribonucleoprotein complexes with different protein contents or different conformations of the same complex. The migration profile was not altered by increasing KCl concentrations, indicating that RNA-protein interactions are stable (data not shown).

To localize a binding region for Orf1, other transcripts were tested (Fig. 5A). mRNAs 2 and 3 start 35 and 53 bp downstream of + 1 transcription initiation point respectively. mRNA 4 starts at the AUG of the *abiD1*-coding region, and thus lacks a translation initiation signal. mRNA 5 starts at the + 1 transcription point and ends at the AUG of the *abiD1 *coding region. The mRNAs 6 and 7 correspond to the internal part of the *abiD1 *gene. Four transcripts (mRNAs 2, 3, 4 and 5) gave results similar to those obtained with mRNA 1 (data not shown). In contrast, Orf1 did not bind transcripts 6 and 7(Fig. 5C). To control specificity of Orf1-RNA binding we used two other *L. lactis *transcripts:*aldB *and *trpA *mRNAs [52,62]. No binding to either RNA was detected (Fig. 5D). These results indicate that Orf1 specifically binds *abiD1 *TIR mRNA in the 5'-region.

### Orf1 might recognize a specific RNA motif

**Figure 6:**
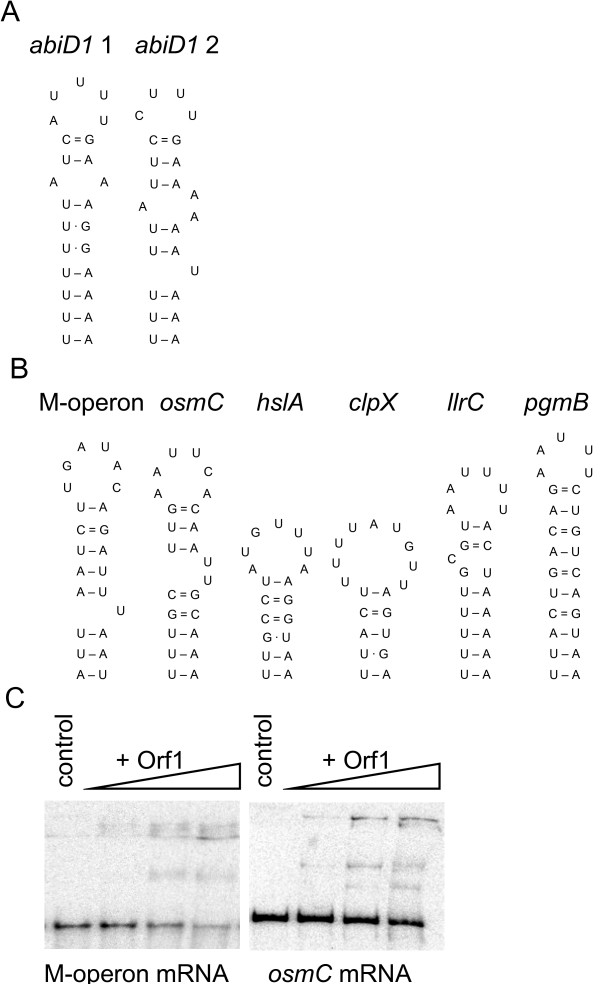
**Putative Orf1-binding mRNA motif**. (A) Predicted mRNA secondary structures reveled by FOLDALIGN [67,68] in *abiD1 *mRNA (nucleotide positions are 7341–7362; 7518–7539; GenBank Accession Number AF116286) and (B) phage bIl66 mid-infection induced M-operon mRNA (nucleotide positions are 566–591; GenBank Accession Number L35175) and *L. lactis *IL1403 cold shock inducible genes *osmC*, *hslA*, *clpX*, *llrC *and *pgmB *mRNAs (nucleotide positions are 68675–68698; 502344–502363; 1163846–1163866; 403740–403763 and 442024–442049, respectively; GenBank Accession Number NC_002662). Gene names are indicated. (C) Binding of Orf1 to M-operon and *osmC *RNAs. Experiments were performed with increasing amounts of Orf1 (0; 0.05 μM; 0.11 μM; 0.22 μM) in the same conditions as above.

To identify putative sequences or structural determinants involved in Orf1 interaction, we analyzed the mRNA transcript sequences used in binding experiments (*abiD1 *mRNAs 1 to 7, *aldB *mRNA and *trpA *mRNA). Initial analyses using BLAST tools did not reveal any conserved sequence pattern in Orf1-binding transcripts. In contrast, FOLDALIGN, a method which detects common stem-loop RNA motifs in unaligned sequences [63,64], revealed the presence of an mRNA stem-loop secondary structure in all *abiD1 *mRNAs shown to bind Orf1 (Fig. 6A). The secondary structures were cross-validated by MFOLD [45]. The putative RNA secondary structures are weak (ΔG = -4.5 and -6 Kcal/mole at 30°C), and are formed by AU-rich RNA (82% and 86%), thus differing from stem-loop structure formed by *L. lactis aldB *TIR GC-rich mRNA (ΔG = -15 Kcal/mole; 45% AU). The structure present in *abiD1 *RNAs 1, 2, 3 and 4 is located upstream of the *abiD1 *ATG codon and sequesters *abiD1 *RBS. The second structure present in RNA 5 is located 152 nucleotides downstream of ATG codon. RNAs that were negative for Orf1 binding (*abiD1 *RNAs 6 and 7, and *aldB *and *trpA *mRNAs) were devoid of such RNA secondary structures.

We hypothesized that these structures could be involved in Orf1-RNA recognition. To verify this, we searched for other mRNAs containing similar structures and tested whether they can bind Orf1. First, we analyzed the sequence of the phage bIL66 mid-infection M-operon coding for Orf1. The *orf1 *gene is known to be involved in the regulation of expression of a phage structure-specific DNA endonuclease, encoded by the same operon [28,29]. The FOLDALIGN method revealed a putative AU-rich RNA secondary structure (ΔG = -4.5 Kcal/mole, Fig. 6B) within the M-operon mRNA, upstream of *orf2 *RBS, which initiates translation of the endonuclease [[29]; our unpublished results]. Moreover, no secondary structures like those above could be detected in 5'-untranslated sequences of two *L. lactis *genes coding for AbiD and AbiF abortive infection proteins, which share 28% to 46% of identity with AbiD1 protein, but are not regulated by Orf1 (GenBank accession numbers AAA63619 and ABG00298 respectively, our unpublished results). We also looked at known *L. lactis *cold-shock inducible genes, *pgmB*, *osmC*, *llrC*, *hslA *and *clpX *[65,66]. Interestingly, FOLDALIGN revealed possible structures similar to those described above, upstream of putative RBS motifs of each of these genes (Fig. 6B). In contrast, no such structures were found in the intragenic regions of *L. lactis *IL1403 biosynthetic *trp *and *his *operons, which are known not to be regulated by low temperature (GenBank accession number NC_002662).

Next, we tested the capacity of Orf1 to bind two mRNAs containing the identified RNA motif: phage M-operon and *L. lactis osmC*. Transcripts were synthesized in *vitro *using phage T7 RNA Polymerase. M-operon transcript started at the + 1 transcription initiation point, was 225 nucleotides long, and included the RBS and the putative stem-loop structure.*L. lactis osmC *transcript started 130 nucleotides upstream of the ATG codon, was 190 nucleotides long, and included the RBS and the putative stem-loop structure. Orf1 was able to bind both *osmC *mRNA and M-operon mRNA (Fig. 6C). Taken together, our results suggest that Orf1 most probably binds mRNA *via *recognition of specific secondary structure motif.

### Orf1 binding alone does not account for *abiD1 *translation activation

To test whether Orf1 binding to the *abiD1 *mRNA is sufficient to activate AbiD1, we purified the two mutant proteins, Orf1M1 and Orf1M2, encoded by AbiD1 resistant phages bIL66M1 and bIL66M2. Although these proteins are unable to activate *abiD1 *translation, they were found to bind *abiD1 *mRNAs 1, 2, 3, 4 and 5 in the same manner as wild-type Orf1 (Fig. 5B, data shown for mRNA 1) and not RNAs 6 and 7 (data not shown). These results indicate that activation of *abiD1 *translation is not due exclusively to Orf1 binding, even if it seems likely that binding plays a role in activation.

## Discussion

Expression of the lactococcal phage abortive infection mechanism AbiD1 is repressed under normal growth conditions and activated following phage infection and during cell growth at low temperature. Phage Orf1 protein and cold shock are shown here to activate expression of the *abiD1 *gene at the level of mRNA translation.

Expression of *abiD1 *is tightly controlled. Transcription of *abiD1 *is weak, and *abiD1 *mRNA is unstable and poorly translated. A putative mRNA stem-loop structure in the *abiD1 *TIR might sequester both the *abiD1 *RBS and an upstream poly-U sequence. Using translational fusions of *abiD1 *TIRs with *luxAB *gene, we showed that this structure had a negative effect on luciferase synthesis. However, even with a free access to the *abiD1 *RBS, translation initiation was inefficient. These data suggest that *abiD1 *full expression might depend on activation. Here we show that the phage bIL66 *orf1 *gene, which is responsible for its sensitivity to AbiD1, activates *abiD1 *translation. In contrast, the *orf1 *genes from AbiD1^R ^mutants do not activate translation. This positive regulation of the AbiD1 phage abortive mechanism is somewhat similar to activation of the *E. coli *exclusion systems Lit and Prr by phage T4 encoded proteins Gol and Stp, respectively, as mutations in the corresponding phage genes abolish activation [11]. Thus, activation of latent Abi mechanisms by phage proteins synthesized during infection seems to be a feature common to different mechanisms. Our study describes the first example of a mechanism in which Abi activation takes place at the level of translation.

Examples of protein-mediated positive regulation of translation, like that described in this paper, are very rare. Those studied are mediated by modification of local mRNA structure, thereby facilitating ribosome access to RBS, or by modification of some components of the cellular transcription-translation apparatus with consequent change in translation of some specific mRNAs [67-69]. Post-transcriptional mechanisms were shown to play a major role in adaptation of vegetative *E. coli *cells to the cold. Preferential translation of cold shock-induced genes at low temperatures is due to *cis*-elements found in the 5'-untranslated region of at least some mRNAs and *trans*-acting factors [70,71]. Activation of the *abiD1 *translation and the consequent abortive infection phenotype by a temperature decrease of 10°C–12°C is of immediate interest, as such a temperature drop was shown to induce a cold-shock response [72,73]. It therefore appears that AbiD1 is a cold shock-inducible protein. This conclusion is supported by the observation that overproduction of the ribosome binding factor RbfA, which is essential for ribosomal adaptation to the cold, prevents induction of *abiD1 *by low temperature.

The detailed mechanism of Orf1 action remains to be established. Binding to a structure found in the 5'-untranslated regions of *abiD1 *and phage M-operon mRNAs could be involved. Nevertheless, the mutant proteins that were unable to activate AbiD1 translation, do bind the motif with comparable efficiency, which indicates that binding alone is not sufficient for activation. We propose that in the course of phage infection Orf1 binds to *abiD1 *mRNA and acts as a "platform" that interacts with additional factors involved in mRNA translation. In this case, mutations would alter the interaction. Similar mechanism was proposed for *E. coli *Hfq protein involving in regulation of translation of *rpoS *gene [74]. Identification of the cellular partner(s) of Orf1 protein should clarify our understanding of Orf1-mediated translational activation of *abiD1 *expression, and the role of the *orf1 *gene in phage development.

## Conclusion

We studied expression of the *abiD1 *gene at the transcriptional and post-transcriptional level. Expression of *abiD1 *is specifically activated at the level of mRNA translation by the phage-encoded Orf1 protein. The loss of ability to activate translation of *abiD1 *mRNA determines the molecular basis for phage resistance to AbiD1. Identification of temperature decrease as an environmental signal activating AbiD1 phenotype indicates that the *abiD1 *is a cold shock inducible gene.

## Authors' contributions

EB conceived and performed the experiments, analyzed the data and drafted the manuscript; AC performed the QRT PCR analysis, participated in Orf1-RNA binding experiments and in revising the manuscript; MCC and SDE participated in the analysis of data and helped draft the manuscript; all authors read and approved the manuscript.

## Supplementary Material

Additional File 1 **Oligonucleotides used in this study.** This table contains a list of the oligonucleotides used in the study. Click here for file

Additional File 2 **Transcriptonal analysis of the abiD1 gene.** This file provides results of Northern hybridization analysis of the *abiD1 *gene region in the presence and in the absence of the *orf1 *gene. A) Schematic organization of the *abiD1 *gene region. Bent arrows and circles denote promoter and terminator sequences, respectively. Position of the oligonucleotide used as probe for Northern hybridization is indicated by tail-less arrow. Transcripts initiated at the *abiD1 *promoter are shown by solid (50 b) and broken (3.6 kb) lines. B) Northern hybridization results. RNA was extracted from IL1403 AbiD1^+^, pIL2002 [28] and IL1403 AbiD1^+^, pIL253 cells grown with Cm. Hybridization was performed with oligonucleotide n°1 as probe. Click here for file
