# Supplementary material for: Activation of mRNA translation by phage protein and low temperature: the case of Lactococcus lactis abortive infection system AbiD1
Source: BMC Mol Biol. 2009 Jan 27;10:4. doi: 10.1186/1471-2199-10-4 (PMC2661086; doi:10.1186/1471-2199-10-4)
Supplement: Additional File 1 — Oligonucleotides used in this study. This table contains a list of the oligonucleotides used in the study. [file 1471-2199-10-4-S1.pdf]

Oligonucleotides used in this study.

N°	sequence (5'-3')[a]	DNA, accession number or reference
1.	GCGCATACAAAGCAGAGG	pIL105, *abiD1*, AF116286
2.	ATGAAAAATAAACAATTACAAAAATAC	pIL105, *abiD1*, AF116286
3.	ATCATAGGAACATCGGTTG	pIL105, *abiD1*, AF116286
4.	GACCGAAGTGATGAAGCG	*luxAB*, [50]
5.	GAGATATGGCATCACATC	*luxAB*, [50]
6.	AGTGTTCGTATTGAAGAAAGTGAGC	pIL105, *abiD1*, AF116286
7.	AATCACAGTTACTCCCTCAATATCC	pIL105, *abiD1*, AF116286
8.	TTCGACAAGCAGAAGACGTAAA	*luxAB*, [50]
9.	TCGGCTGTTATCCATGTCTGTA	*luxAB*, [50]
10.	GAACACATCTTGCTTTCACGTC	*L. lactis* IL1403, *tuf*, NC002662
11.	AGAGGTCACGAACTTCCATTTC	*L. lactis* IL1403, *tuf*, NC002662
12.	ACTTGCATGTGTTAGGC	*E. coli* 16 rRNA, NC 000913
13.	CG**GGATCCCATATG**ATTTTTTCCTTC-AAAATGATAAAA	pIL105, *abiD1*, AF116286
14.	CG**GAATTC**ATCAGTACTTGCCACGCC-TCTG	pIL105, *abiD1*, AF116286
15.	CG**GAATTC**TATGGCCAGGCTTTTTTTTATC	pIL105, *abiD1*, AF116286
16.	CG**GAATTC**TTTTATCATTTTGAAGGAAAAA-ATATG	pIL105, *abiD1*, AF116286
17.	CG**GAATTC**TTGAAGGAAAAAATCATATG	pIL105, *abiD1*, AF116286
18.	CC**ATCGAT**GGGCGAATTGGAGCT	pIL105, *abiD1*, AF116286
19.	CG**GAATTC**GCATACAAAGTCAATCATAG	bIL66 M-operon, *orf1*, L35175
20.	CG**GAATTC**GTTTCTTTATTCTCCTTTAAAA-ATT	bIL66 M-operon, *orf1*, L35175
21.	CG**GAATTC**CTATTTTCCCATTAATATTTTC-	*L. lactis* IL1403, *aldB*, NC002662

	TA	
22.	CG**GGATCCCATATG**GATATTTCTCTTTT-CTATCTC	*L. lactis* IL1403, *aldB*, NC002662
23.	GCGC**TTAATTAA**CAT**TAGGAAGGAGCG-**TTTCTTTAATGACAGAAGAACAGCTAC	bIL66 M-operon, *orf1*, L35175
24.	CG**GGATCC**TTTATTCTCCTTTAAAAATTA	bIL66 M-operon, *orf1*, L35175
25.	ACGC**GTCGAC**ATAGGAGGAGAAAATGG	*L. lactis* IL1403, *rbfA*, NC002662
26.	AAA**CTGCAG**ATTAATATTCTGATTTATT-GTTAAGCC	*L. lactis* IL1403, *rbfA*, NC002662
27.	GGAATTC**CATATG**ACAGAAGAACAGC	bIL66 M-operon, *orf1*, L35175
28.	CG**GGATCC**CATTTCTTTATTCTCCTT	bIL66 M-operon, *orf1*, L35175
29.	GTGATCT**CATATG**GACTACAAAGACCA-TGACG	p3×FLAG-CMV-7 vector (Sigma-Aldrich)
30.	CGGTACC**CATATG**GTCATCGTCATCCTT-GTAATCG	p3×FLAG-CMV-7 vector (Sigma-Aldrich)
31.	GGTGGTT**GCTCTTCC**AACATGACAGAAG-AACAGCTACTA	bIL66 M-operon, *orf1*, L35175
32.	GGTGGTT**GCTCTTCC**AACATGGTTGACT-TTAACGAGTTC	bIL66 M-operon, *orf1*, L35175
33.	TAA**GAATTC**TTATTATTCTCCTTTAAAA-ATTAAATCTG	bIL66 M-operon, *orf1*, L3517
34.	GGATCCTAATACGACTCACTATAGGG-ATCAGTACTTGCCACGCC	pIL105, *abiD1*, AF116286
35.	GGATCCTAATACGACTCACTATAGGG-TATGGCCAGGCTTTTTTTTTATC	pIL105, *abiD1*, AF116286
36.	GGATCCTAATACGACTCACTATAGGG-TTTTATCATTTTGAAGGAAAAAAT	pIL105, *abiD1*, AF116286
37.	GGATCCTAATACGACTCACTATAGGG-ATGAAAAATAAACAATTACAAAAATAC	pIL105, *abiD1*, AF116286
38.	GGATCCTAATACGACTCACTATAGGG-TTGATAAAAGTGTTCGTATTGAAGAAGT-GACCTCGC	pIL105, *abiD1*, AF116286

39. GGATCCTAATACGACTCACTATAGGG-AGCCTATGAAAAGCTGGATAACCG	pIL105, *abiD1*, AF116286
40. GCGAGCTCATCACTTTCTTCAAT	pIL105, *abiD1*, AF116286
41. CATATGATTTTTTCCTTCAAAATGATAAAA	pIL105, *abiD1*, AF116286
42. CAATCACAGTTACTCCCTCAAT	pIL105, *abiD1*, AF116286
43. ATCATAGGAACATCGGTTG	pIL105, *abiD1*, AF116286
44. GGATCCTAATACGACTCACTATAGGG-AAGTAGAAAATCAATTTGCTAAGTATTC	*L. lactis* IL1403, *osmC,* NC002662
45. GCCAAAACCATTTTCTAAAG	*L. lactis* IL1403, *osmC,* NC002662
46. GGATCCTAATACGACTCACTATAGGG-TATTTTCCCATTAATATTTTCTAGTC	*L. lactis* IL1403, *aldB,* NC002662
47. GTTAGTTAAGAGGTAGTTGTC	*L. lactis* IL1403, *aldB,* NC002662
48. GGATCCTAATACGACTCACTATAGGG-CTTGAAGCAGAAAAAGAGGTG	*L. lactis* IL1403, *trpA,* NC002662
49. GGAACGCCAATTTCAATAGCG	*L. lactis* IL1403, *trpA,* NC002662
50. GGATCCTAATACGACTCACTATAGGG-ATCAAGTTAAGAGAGGAAAGC	bIL66 M-operon, L35175
51. GCTTAACATAGTGTTCGTAC	bIL66 M-operon, *orf1*, L35175

[a] Extensions containing restriction sites are shown in bold; T7 promoter sequence is underlined.
