# Supplementary figures and images for: Activation of mRNA translation by phage protein and low temperature: the case of Lactococcus lactis abortive infection system AbiD1
Source: BMC Mol Biol. 2009 Jan 27;10:4. doi: 10.1186/1471-2199-10-4 (PMC2661086; doi:10.1186/1471-2199-10-4)

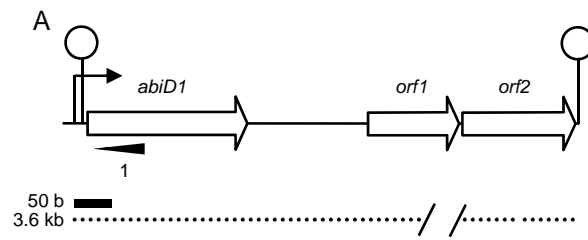

B

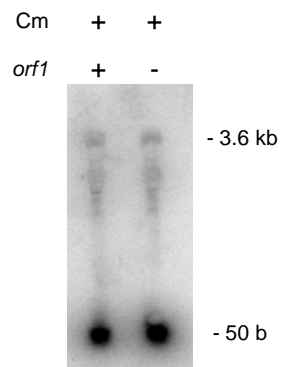

Supplement: Additional File 2 — Transcriptonal analysis of the abiD1 gene. This file provides results of Northern hybridization analysis of the abiD1 gene region in the presence and in the absence of the orf1 gene. A) Schematic organization of the abiD1 gene region. Bent arrows and circles denote promoter and terminator sequences, respectively. Position of the oligonucleotide used as probe for Northern hybridization is indicated by tail-less arrow. Transcripts initiated at the abiD1 promoter are shown by solid (50 b) and broken (3.6 kb) lines. B) Northern hybridization results. RNA was extracted from IL1403 AbiD1+, pIL2002 [28] and IL1403 AbiD1+, pIL253 cells grown with Cm. Hybridization was performed with oligonucleotide n°1 as probe. [file 1471-2199-10-4-S2.pdf]
